# Craniofacial growth predictors for class II and III malocclusions: A systematic review

**DOI:** 10.1002/cre2.357

**Published:** 2020-12-04

**Authors:** Antonio Jiménez‐Silva, Romano Carnevali‐Arellano, Sheilah Vivanco‐Coke, Julio Tobar‐Reyes, Pamela Araya‐Díaz, Hernán Palomino‐Montenegro

**Affiliations:** ^1^ Orthodontic and Orthopaedic Department, Faculty of Dentistry Universidad Andrés Bello Santiago Chile; ^2^ Department of Prosthodontics, Faculty of Dentistry University of Chile Santiago Chile

**Keywords:** class II malocclusion, class III malocclusion, growth and development, growth predictors

## Abstract

**Objective:**

To evaluate the validity of craniofacial growth predictors in class II and III malocclusion.

**Material and methods:**

An electronic search was conducted until August 2020 in PubMed, Cochrane Library, Embase, EBSCOhost, ScienceDirect, Scopus, Bireme, Lilacs and Scielo including all languages. The articles were selected and analyzed by two authors independently and the selected studies was assessed using the 14‐item Quality Assessment Tool for Diagnostic Accuracy Studies (QUADAS‐2). The quality of evidence and strength of recommendation was assessed by the GRADE tool.

**Results:**

In a selection process of two phases, 10 articles were included. The studies were grouped according to malocclusion growth predictor in (1) class II (*n* = 4); (2) class III (*n* = 5) and (3) class II and III (*n* = 1). The predictors were mainly based on data extracted from cephalometries and characterized by: equations, structural analysis, techniques and computer programs among others. The analyzed studies were methodologically heterogeneous and had low to moderate quality. For class II malocclusion, the predictors proposed in the studies with the best methodological quality were based on mathematical models and the Fishman system of maturation assessment. For class III malocclusion, the Fishman system could provide adequate growth prediction for short‐ and long‐term.

**Conclusions:**

Because of the heterogeneity of the design, methodology and the quality of the articles reviewed, it is not possible to establish only a growth prediction system for class II and III malocclusion. High‐quality cohort studies are needed, well defined data extraction from cephalometries, radiographies and clinical characteristics are required to design a reliable predictor.

## 1 INTRODUCTION

The precision in the diagnosis and evaluation of growing patients is relevant in the field of orthodontics, since it allows the prediction and assessment of the amount of growth for planning orthopedic, orthodontic or surgical treatment (Alexander et al., 2009) with the aim of a successful outcome.

The great variability in the direction and quantity of craniofacial growth implies great importance for success in orthodontic treatment, which has generated great interest in the search for methods of predicting individual facial growth in terms of direction and magnitude (Solow & Siersbaek‐Nielsen, 1992), since it would allow to estimate future changes in the vertical or horizontal relationship (Turchetta et al., 2007).

In the past, the theory popularized by Brodie (1941, 1946) and Brodie et al. (1938) indicated that growth patterns were established at an early age; however, evidence would show that there are changes in the growth pattern over time in both direction and quantity, which would support the search for some system to predict craniofacial growth in the future (Rudolph et al., 1998). The interaction between all components of the craniofacial system, such as genetic and environmental factors (Auconi et al., 2014), increases the complexity of their growth prediction. Therefore, the integration of the components should be established to obtain predictive models developed in recent times and that have allowed us to infer the progression of the dentoalveolar imbalance congruent with the biological principles of growth and development (Araya‐Díaz et al., 2013; Auconi et al., 2014; Janes & Yaffe, 2006; Ruz & Araya‐Díaz, 2018).

Among the different prediction methods available for craniofacial growth, there are systems based on statistical information according to averages of growth increments (Solow & Siersbaek‐Nielsen, 1992); Another approach uses facial structure characteristics: Facial types, structural features of lower face (Lavergne, 1982), n‐tgo gn angle, proportion of anterior to posterior facial height (Solow & Siersbaek‐Nielsen, 1992), regression equations to predict mandibular rotation (Skieller et al., 1984), graphic projection techniques (Ricketts, 1972), cervical and craniofacial posture (Solow & Siersbaek‐Nielsen, 1992) and development of mathematical models from computational techniques extracted from cephalometric data (Auconi et al., 2014), cephalometric criteria and procedures such as meshing criteria, grids among others (Johnston, 1975; Moorrees & Lebret, 1962; Popovich & Thompson, 1977; Ricketts, 1972). Despite the existence of these predictors, there would not be methods with relevant clinical acceptability to predict growth (Hirschfeld & Moyers, 1971), which makes it difficult to generate a proposal for use in orthodontic practice.

Pre‐adolescent subjects with class II malocclusion have favorable and unfavorable growth patterns and their predictability could determine the planning and result of orthodontic treatment (Rudolph et al., 1998). Despite the characterization of these patients, there is no precise method to predict the amount, direction and magnitude of their growth, as it would be difficult to determine the contribution of the predictors when craniofacial changes occur due to treatment or growth.

In subjects with class III malocclusions, evidence based on longitudinal studies would indicate differences in mandibular growth compared to class I subjects, where skeletal and dental components tend to manifest early in class III children (Guyer et al., 1986; Tollaro et al., 1994) and they would worsen with growth (Alexander et al., 2009). Reyes et al., 2006, indicate that there would be no tendency for sagittal self‐limitation in class III malocclusions (Reyes et al., 2006). In addition, there would be multiple environmental, behavioral and genetic factors contributing to the determination of mandibular morphology and where genetic factors would play a significant role (Bayram et al., 2014; Huh et al., 2013). This multifactorial characteristic would make it more difficult to establish a prediction system.

The purpose of this study was to identify and analyze prediction methods to determine growth in subjects with class II and III malocclusions to estimate skeletal, sagittal and vertical dentoalveolar changes.

## 2 MATERIALS AND METHODS

This systematic review was conducted according to the Preferred Reporting Items for Systematic Reviews and Meta‐analyses (PRISMA) statement (Moher et al., 2009).

The aim of this systematic review was to answer the PICO question (Population, Intervention, Control groups and Outcome): “What are the prediction methods (I) to accurately determine the growth in the short and long term (O) in patients with class II and III malocclusion (P) when comparing craniofacial growth over time (C)?” an electronic search was conducted in April 2019, updated on 23 August 2020. The electronic databases used were PubMed, Cochrane Library, Embase, Scopus, EbscoHost, ScienceDirect, Bireme, Lilacs y Scielo.

## 3 STUDY SELECTION

### 3.1 Inclusion criteria for this review were as follows:

#### 3.1.1 Types of studies

Cohort studies with the objective of designing or proposing some method to predict growth in patients with skeletal class II and III malocclusion.

#### 3.1.2 Language of the studies

Search of studies without limitation of language, but the studies included for analysis were in Spanish, English and Portuguese. This is based on the fact that these are the languages used by researchers.

#### 3.1.3 Types of participants

Selected studies included growing subjects of both genders, with the clinical/imaging diagnosis of skeletal class I, II, and III malocclusions. The participants included were not subjected to a surgical procedure in the facial skull region, were not subjected to any previous orthopedic or orthodontic treatment and nor did they present any syndrome or alteration of facial skull growth.

#### 3.1.4 Intervention types

Studies without intervention, with the aim of designing and proposing predictors of growth in the short and long term in subjects with class II and III malocclusion.

### 3.2 Types of results

**Primary outcomes:** Analyze studies that design and propose predictors of vertical and/or sagittal growth in growing subjects with class II and III malocclusions from clinical or imaging data, analyze the available evidence when determining the cephalometric or clinical predictors constructed using computational modeling, mathematical equation and other methods based on statistical analysis. In addition, establish the risk of biases of these studies to determine their methodological quality.

### 3.3 Data collection

**For class II and III diagnostic:** Data obtained from cephalometric methods (Steiner, Ricketts, Delaire analysis among others), radiographs for orthodontic planning. Clinical methods (occlusal, intraoral and extraoral examination), laboratory (biological samples analysis) and methods based on mathematical models with data obtained from clinical, imaging and/or cephalometric data.

**Predictor construction:** Multivariate or univariate analysis, computational methods (based on discriminant analysis, machine learning), mathematical modeling among others.

### 3.4 Search strategy

For the identification and selection of the number of potentially eligible studies for this systematic review (N), a specific and individualized search strategy was developed for each database. A semantic field was determined for the term “Class II and III malocclusion” and another semantic field related to the term “Growing Predictors.” The search strategy is found in Table A1 in Appendix of this review.

### 3.5 Study selection

In a first screening, the title and abstract of all potentially eligible articles were listed and evaluated by two researchers independently (J.A., C.R.). In a second stage, the full text of articles that potentially met eligibility criteria based on the first screening was assessed independently by the same two researchers (J.A., C.R.) according to inclusion criteria (study design: clinical trial, diagnostic studies; objective: to propose predictors based on clinical, imaging, cephalometric methods, mathematical models among others, that allow to predict growth for class II and III patients; type of participants: patients in the growth stage). When no agreement was found, the inclusion of the article within the sample was discussed with a third researcher (A.P.) who acted as an arbiter. Articles that met inclusion criteria were included in the review for the final analysis. The reasons why some studies were excluded were recorded in an adjacent column (Table A2 in Appendix). The quality of assessment according to GRADE, was performed by two independent reviewers (V.S. and T.J.). To determine the quality and methodological validity in relation to the diagnostic methods of the selected studies, Quality assessment of studies of diagnostic accuracy included in Systematic Reviews – QUADAS‐2 was used (V.S. and T.J.).

### 3.6 Extracting data from studies and data synthesis

The PICO format (Population, Intervention, Control groups and Outcome) was used to make the tables of analyzed articles: Population (sample size, distribution by gender, age range and SD); Intervention: (Instrument for malocclusion diagnostic, image acquisition protocol and type of predictor); Comparison criteria or control: (comparison of craniofacial growth over time) and Outcomes (including the answer to the hypothesis, statistical analysis. Finding overall).

### 3.7 Risk of bias in individual studies

The Grading of Recommendations Assessment, Development and Evaluation (GRADE) system (GRADE, 2014), was used to evaluate the quality of evidence. Two authors independently assessed the quality of the evidence and the strength of the recommendations according to the risk of bias. The methodological quality of the selected studies was evaluated with the QUADAS‐2 tool (Whiting et al., 2011), used to assess the quality of diagnostic accuracy studies. Two authors independently rated each item as “yes,” “no,” “unclear,” “low” or “high.”

## 4 RESULTS

**FIGURE 1:**
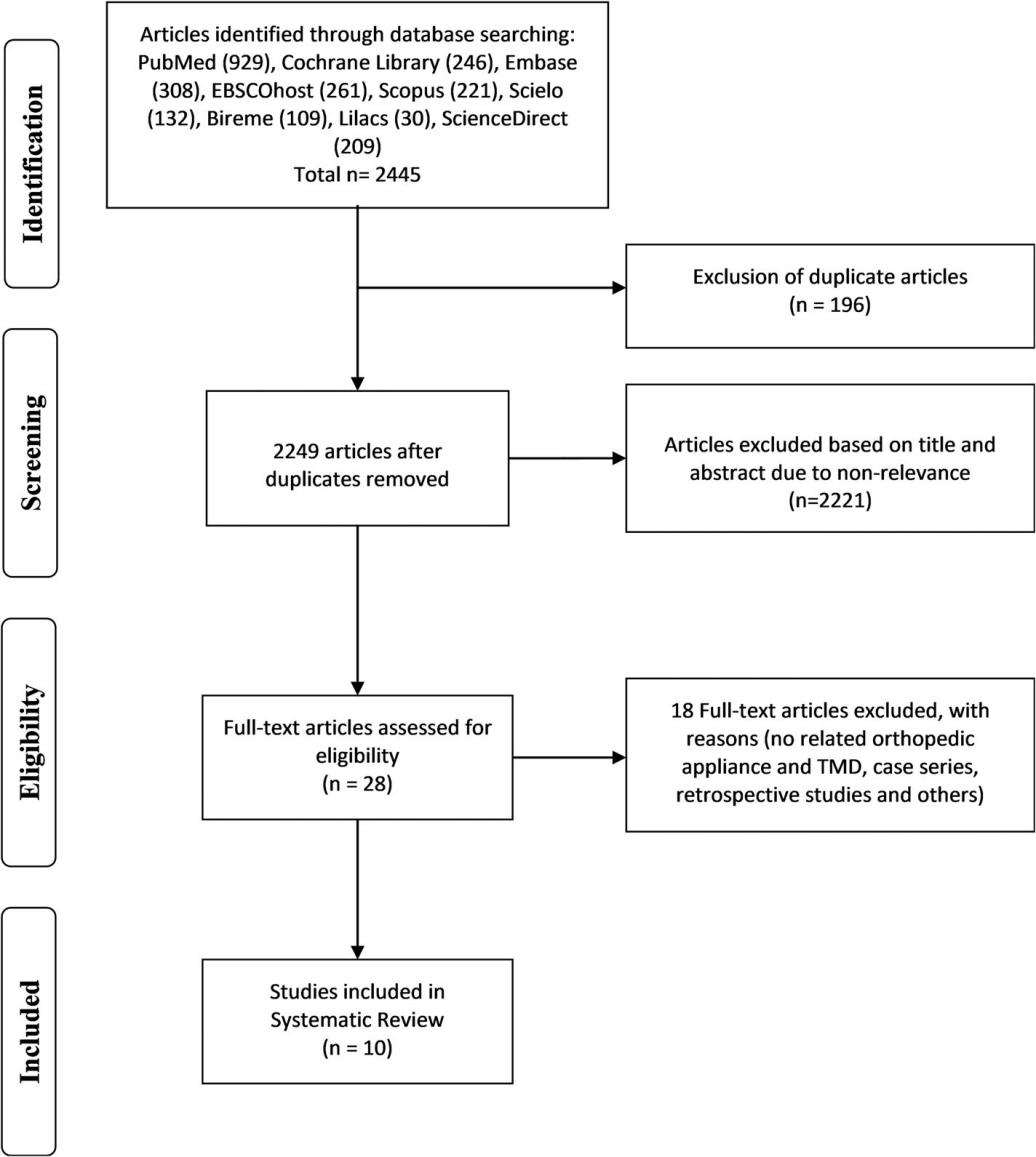
Search method, identification, selection and inclusion of articles. PRISMA flow diagram

2445 articles were identified from the 9 electronic databases. The studies were exported to an‐Excel table, and of these articles, 196 were eliminated because they were duplicates. The remaining 2249 studies were evaluated by the authors in a first screening and 2221 of these were eliminated because they were not relevant for this study. Of the remaining 28 studies, 18 were eliminated in a second screening when the full text of the articles was analyzed, and the reasons for exclusion are shown in Table A2 in Appendix. Finally, 10 studies were analyzed qualitatively. The search results are presented in Table A1 in Appendix and the flowchart of the literature search is presented in Figure 1.

### 4.1 Study characteristics

#### 4.1.1 Characteristics of participants

In the articles analyzed (Tables 1, 2, 3), a total of 1313 participants were investigated, with an age range between 6 to 20 years, both genders were included, although three studies included only female subjects (Auconi et al., 2014; Chen et al., 2005; Scala et al., 2012) and 1 only male (Buschang et al., 1986). According to the type of malocclusion, the studies analyzed included class II skeletal malocclusion (*n* = 4); class III (*n* = 5) and class II/III (*n* = 1) (Table 4).

**TABLE 1:** Summary of studies that analyzed growth predictors in class II malocclusion (*N* = 4)

	Population		Intervention		Comparison		Outcome		
First author and year	Number of subjects	Age (mean age/range) and gender	Malocclusion diagnostic instrument	Predictor	Study group	Control group	Statistical analysis or mathematical model	Overall findings	Conclusions
Arias et al. (2006)	24	16 male and 8 females; age range 6–17 yrs	Cephalometric analysis (linear and angular measurements).	Mathematical equation: *p* = P(*Y* = 1) = 1/1+ ℯ^(38.4199–0.1849*X*^_1_^‐0.8084*X*^_*2*_^+ 0.4945*X*^_3_^– 0.6776*X*^_4_^)^	Class II	Class I	Multivariate analysis using logistic regression	Prediction level was 95.7% with greater sensitivity. Sensitivity to detect class II subjects was 70.6%.	The variables SNA, CO‐A, Co‐GN and ANB have a 99% prediction for the development of a class I malocclusion and 71% for class II.
Rudolph et al. (1998)	31	19 girls and 12 boys	Cephalograms (linear, angular or proportional) 48 measurements at 6, 8, 10, and 12 years.	Growth prediction formulas: 1. P (Good | Fn) = k1e –(0.5) | Fn ‐ μng |∑g −1 | Fn ‐ μng | T 2. P (Poor | Fn) = k2e –(0.5) | Fn ‐ μnp |∑p −1 | Fn ‐ μnp | T	Group 1 (poor growers): 20.	b) Group 2 (good growers): 11.	Bayes' theorem. Dahlberg's formula.	Prediction equations to differentiate between good and poor growth patterns of skeletal class II preadolescents was 91% accurate	Multivariate growth prediction equations presented can be used to successfully predict patterns of growth in skeletal class II patients.
Solow and Siersbaek‐Nielsen (1992)	34	16 girls and 18 boys. Mean age 9.9 years at time 1 and 12.7 years at time 2.	Cephalograms.	Cephalometric and hand‐wrist radiographs. Mean duration of the observation period was 2.8 years (SD 0.4, range 2.0 to 3.6 years).	Class II,1:18 Class II,2:4	Class I malocclusions: 12	Correlation coefficients.	Craniofacial growth were found between cervical and craniocervical posture and sagittal displacement of articulare (n‐ar, n‐s‐ar), maxillary growth in length (ss‐pm), change in facial prognathism (s‐n‐ss, s‐n‐sm, s‐n‐pg), and rotation of the mandible(NSL/REFm1, REFcrb/REFml).	There is a relationship between craniocervical posture in prepubertal children and the direction of facial development
Buschang et al. (1986)	40	No information.	Cephalometric analysis.	Polynomial model for 15 measures Y = a + bT + u.	Class II malocclusion (*n* = 20)	Normal occlusion (*n* = 20)	MANOVA test	Children with normal occlusion and those with malocclusion are comparable for 80% of their measures. Ba‐Na display significant growth (velocity) differences. Ar‐Po significantly shorter (2.5 mm) in untreated class II.	Polynomial approach provides an important method for describing and evaluating longitudinal craniofacial growth. Polynomial models provide growth estimates describing mean size, velocity, and acceleration

**TABLE 2:** Summary of studies that analyzed growth predictors in class III malocclusion (*N* = 5)

	Population		Intervention		Comparison		Outcome		
First author and year	Number of subjects	Age (mean age/range) and gender	Malocclusion diagnostic instrument	Predictor	Study group	Control group	Statistical analysis or mathematical model	Findings overall	Conclusions
Auconi et al. (2014)	429	Female. 7 years 2 months to 17 years 3 months.	Cephalometric analysis. Clinical criteria.	Computational modeling: Network and fuzzy cluster analysis to characterize three distinct class III phenotypic groups.	Independent semi‐longitudinal sample of untreated class III malocclusion. 1. Group G1 (from 7 to 10 years) 195 patients. 2. Group G2 (from 11 to 12 years) 135 patients 3. Group G3 (from 13 to 14 years) 105 patients 4. Group G4 (from 15 to 17 years) 99 patients.		ANOVA. Pearson correlation coefficient	Four parameters provided the best phenotypic grouping of patients: Co‐A, co‐Gn, SNB, and P22 (a combination of SN‐GoGn and ArGoMe angles).	1. A facial pattern already well‐established at the age of 7–9 years maintains the same characteristics in the course of development, once it is framed in the correct reference system. 2. There is a mathematical basis that links the orthodontic auxological laws, the biomechanical laws, and the laws of the auto‐organizing processes
Scala et al. (2012)	532	Female. 6 years 4 months to 17 years 3 months	Cephalometric analysis. Clinical criteria.	Apply conjunctly statistical analysis with network tools. Correlation matrices were analyzed among them by using complex networks.	Untreated Class III Caucasian patients.		Analyze the correlation matrices among cephalographic Landmarks. Pearson correlation coefficient.	The most‐connected nodes are those related to vertical skeletal features (N‐Me, SNGoGn, PP‐PM) and these can be regarded as the key features in the growth of female Class III subjects.	During the growth process of Class III malocclusion the skeletal vertical and sagittal growth features (SN‐GoGn, PP‐PM) are central in the interacting network of the system components. A substantial portion of the Class III issues during growth is driven by only a few nodes.
Chen et al. (2005)	44	Female. Age range 8–18 years.	Cephalometric analysis.	Hand‐wrist and cephalometric radiographs Mandible GP (mm) = 61.01–1.31 × AH3–1.25 × PH3–0.73 × AP3–1.68 × AH4	Group A: 22 girls whose GP (mm) is calculated = Ar‐Pog (final) – Ar‐Pog (initial)	Group B: 22 girls. Used GPM, the MM and the equation to predict mandible GP and then compared with the GP at present	Multiple regression analysis and student's *t* test to compare the predictive accuracy.	The average error of SPE (special method of prediction) was the smallest, Whereas the average error of GPM was the largest. The accuracy of SPE had significant differences compared with the GPM and MM.	The equation might be one possible method for predicting the mandible GP based only on a single cephalometric radiograph.
Abu Alhaija and Richardson (2003)	115	59 females and 56 males. Mean age: 11.6 ± 1.7 years for females and 12.7 ± 1.3 years for males.	Cephalometric analysis. Clinical criteria.	The stepwise discriminant analysis using the method of Wilks. Linear equation.	Class III malocclusion.		ANOVA and discriminant function analysis.	**First cluster** (*N* = 36): Horizontal discrepancy cases. **Second cluster** (*N* = 26): Intermediate between the first and third clusters. **Third cluster** (*N* = 37): Long face type of Class III. The sum of the deviations of the measurements of molar relation, cranial deflection, ramus position, and porion location were significant in cases with greater mandibular growth ratio.	Discriminant analysis on the initial radiographs of 115 untreated subjects who subsequently grew favorably or unfavorably produced correct outcome prediction in 80% of subjects.
Schulhof et al. (1977)	14	Age range: 6.4 to 12.9 years old.	Cephalometric analysis.	Prediction program: Rocky Mountain data systems and the standard computer program designed for the Japanese race	Skeletal class III.	Japanese in Hawaii modified data based upon the work of Sassouni for the Chinese.	Sum of deviations from normal of the predictor measurements. Growth ratio.	The sum of the deviations of the measurements of molar relation, cranial deflection, ramus position, and porion location were significant in cases with greater mandibular growth ratio.	Four significant factors have been identified in the lateral cephalometric head film which would indicate the likelihood of the patient growing in an abnormal Class III manner

**TABLE 3:** Summary of studies that analyzed growth predictors in class II and III malocclusion (*N* = 1)

	Population		Intervention		Comparison		Outcome		
First author and year	Number of subjects	Age (mean age/range) and gender	Malocclusion diagnostic instrument	Predictor	Study group	Control group	Statistical analysis or mathematical model	Findings overall	Conclusions
Turchetta et al. (2007)	50	Age range: 9–20 years. 26 female and 24 males.	Cephalometric analysis.	Ricketts analysis, the Johnston grid system, and the Fishman system of skeletal maturation assessment	Class I, II, and III malocclusions.		Paired t test	Long‐term prediction of growth was valid in Johnston maxillary and mandibular angular predictions for both sexes and male mandibular linear estimations; Ricketts linear female maxillary and mandibular predictions for both sex subgroups. All Fishman maxillary and mandibular angular measurements, and Class I and III linear measurements were significantly predicted.	The Fishman prediction system adds more individuality than any other systems; it bases its prediction on skeletal maturation determined by an evaluation of the hand‐wrist radiograph. It has been shown that individualizing prediction by assessing maturational development rather than chronologic age can greatly increase the accuracy of prediction.

Abbreviations: Ar‐Po, linear distance from Ar to Po; ArGoMe, Gonial angle; ANB, anteroposterior relation of the maxilla and mandible; Ba‐Na, cranial base length from Ba to Na; Co‐A, midfacial length as distance from Co to A; Co‐Gn, mandibular length as distance from Co to Gn; GP, mandibular growth potential; GPM, mandibular growth potential method; MM, method of Mito et al; N‐Me, anterior facial length; PP‐PM, inclination of the palatal plane in relation to the mandible plane; SD, standard deviation; SNA, anteroposterior maxillary position to the anterior cranial base; SNB, anteroposterior mandibular position to the anterior cranial base; SN‐GoGn, divergence of the mandibular plane relative to the anterior cranial base; SPE, special method of prediction; yrs, years.

**TABLE 4:** Summary of articles included in the analysis according to design, type of malocclusion and predictor

First author and year	Study design	Type of malocclusion	Type of predictor (clinic, imagenologic, laboratory, mathematical models)	Predictive model for maxillary and/or mandibular growth
Auconi et al. (2014)	Cohort study	Class III	**Computational modeling:** Cephalometric and mathematical methods (Network and Fuzzy cluster analysis)	For mandible: Co‐A, Co‐Gn, SNB, and P22 (a combination of SN‐GoGn and ArGoMe angles).
Scala et al. (2012)	Cohort study (retrospective)	Class III	**Network modeling:** Cephalometric, mathematical and software methods (Network analysis and Ed software)	Vertical skeletal features (N‐Me, SNGoGn, PP‐PM)
Turchetta et al. (2007)	Cohort study	Class II and III	**Cephalometric, hand‐wrist radiographs** Ricketts analysis, Johnston grid system, and Fishman	Fishman method: T1‐T2/T2‐T3 and T1‐T3: CC‐A CC‐Gn CCNA CCNGn
Arias et al. (2006)	Cohort study	Class II	**Mathematical equation** From cephalometric data.	SNA, CO‐A, CO‐GN and ANB variables. (*p* = P(*Y* = 1) =1/1+ ℯ^(38.4199–0.1849*X*^_1_^‐0.8084*X*^_*2*_^+ 0.4945*X*^_3_^– 0.6776*X*^_4_^)^
Chen et al. (2005)	Cohort study	Class III	**Linear equation:** Cephalometric, CVMS, hand‐wrist radiographs, and mathematical model	Ar‐Pog (final) – Ar‐Pog (initial). (mandible GP (mm) = 61.01–1.31 x AH3–1.25 x PH3–0.73 x AP3–1.68 x AH4)
Abu Alhaija and Richardson (2003)	Cohort study	Class III	**Cluster analysis (discriminant function analysis)** Cephalometric data.	**D = C + B1X1 + B2X2 + …. +** **BpXp**
Rudolph et al. (1998)	Cohort study	Class II	**Mathematical equation:** From cephalometric data.	ANB angle and its capacity of improvement through the years. (1. P(Good | Fn) = k1e –(0.5) | Fn ‐ μng |∑g −1 | Fn ‐ μng | T 2. P(Poor | Fn) = k2e –(0.5) | Fn ‐ μnp |∑p −1 | Fn ‐ μnp | T)
Solow and Siersbaek‐Nielsen (1992)	Cohort study	Class II, 1 and 2	**Computerized structural superimposition** From cephalometric and hand wrist radiographs.	Maxillary growth in length (ss‐pm) Change in facial prognathism (s‐n‐ss, s‐n‐sm, s‐n‐pg)
Buschang et al. (1986)	Cohort study	Class II Div.1 and 2	**Mathematical model** (Orthogonal polynomial based on 15 cephalometric measurements from cephalometric data).	Linear Growth for maxillary measures (stable relationship with cranial base). Mandibular length (Ar‐Po)/ Length of ramus height (Ar‐Go)
Schulhof et al. (1977)	Cohort study (Retrospective)	Class III	**Software methods:** From cephalometric and clinical data. (Rocky Mountain Data Systems and the standard computer program designed for the Japanese race)	Molar relation, cranial deflection, ramus position, and porion location. = V‐CN × V SD

#### 4.1.2 Characteristics of predictors

All studies included predictors designed from cephalometric data obtained from growing patients and considered only cohort study designs (Table 4). For class II malocclusions, 4 articles were analyzed and the proposed predictors consisted of mathematical equation (Arias et al., 2006; Rossouw et al., 1991; Rudolph et al., 1998) and computerized structural superimposition (Solow & Siersbaek‐Nielsen, 1992). For class III malocclusions, six studies were identified. In these studies, the predictors used were: network and computational modeling (Auconi et al., 2014; Scala et al., 2012), cluster analysis (Abu Alhaija & Richardson, 2003), linear equation (Chen et al., 2005), software methods (Schulhof et al., 1977) and predictive method based on cephalometric analysis (Rossouw et al., 1991). Among the predictors for class II/III malocclusions, only 1 study was found in which they compared Ricketts analysis, the Johnston grid system, and the Fishman method (Turchetta et al., 2007).

#### 4.1.3 Risk of bias of included studies

**FIGURE 2:**
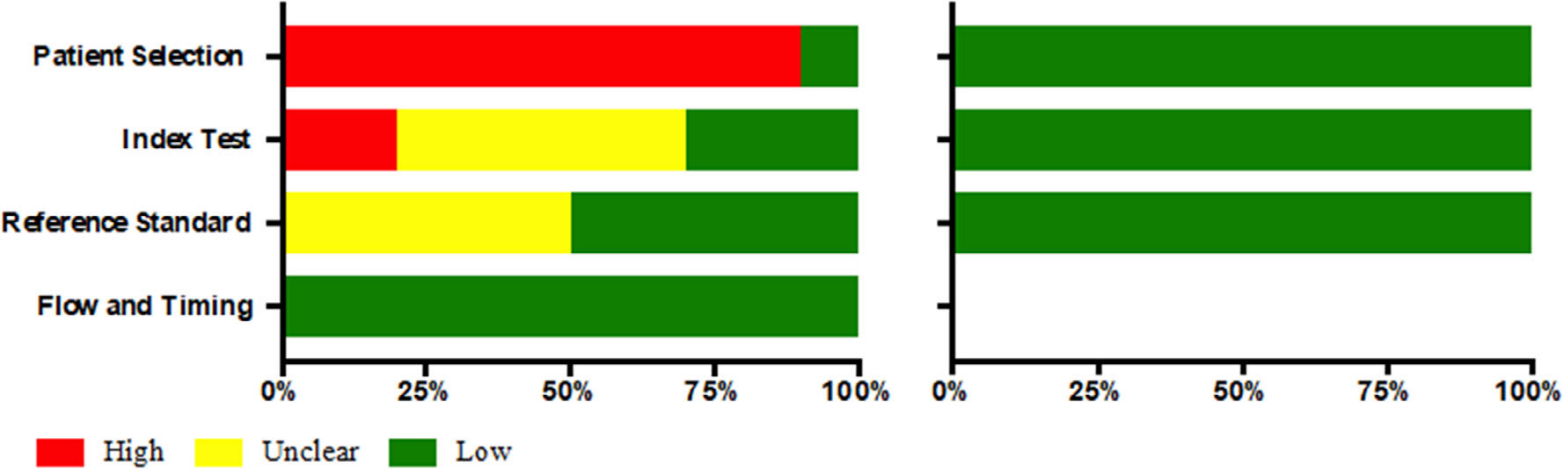
Criteria met, according to the QUADAS‐2 tool

**FIGURE 3:**
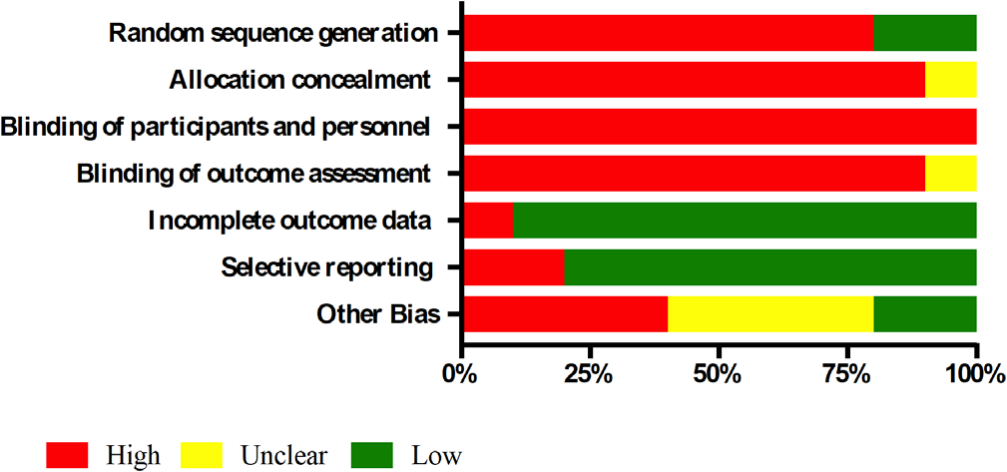
Criteria met, according to the GRADE tool

The studies in general were methodologically heterogeneous, because the types of analysis differed among the included studies, although they all proposed growth predictors from cephalometric and/or clinical data. The methodological quality of the predictors according to QUADAS‐2 was low to moderate and none of the articles met all its criteria (Table 5, Figure 2). The domains with possible bias were patient selection, index test and reference standard. In general, most of the studies presented a high risk of bias in the Patient Selection domain, since there was no randomization of the sample in a large part of the studies analyzed (Abu Alhaija & Richardson, 2003; Arias et al., 2006; Auconi et al., 2014; Chen et al., 2005; Rudolph et al., 1998; Scala et al., 2012; Schulhof et al., 1977; Solow & Siersbaek‐Nielsen, 1992). Bias was observed in the interpretation of the index test (domain 2) (Auconi et al., 2014; Buschang et al., 1986; Chen et al., 2005; Scala et al., 2012; Solow & Siersbaek‐Nielsen, 1992; Turchetta et al., 2007) and also in the lack of clear description in the blinding of researchers when the test results were interpreted (Abu Alhaija & Richardson, 2003; Arias et al., 2006; Auconi et al., 2014; Buschang et al., 1986; Scala et al., 2012; Solow & Siersbaek‐Nielsen, 1992; Turchetta et al., 2007). The analysis of the quality of the evidence, according to the GRADE tool (Table 6, Figure 3) indicated that the available evidence regarding growth predictors in patients with class II and III malocclusions was low.

**TABLE 5:** QUADAS‐2 criteria fulfilled

	Item	Auconi et al. (2014)	Scala et al. (2012)	Turchetta et al. (2007)	Arias et al. (2006)	Chen et al. (2005)	Abu Alhaija and Richardson (2003)	Rudolph et al. (1998)	Solow and Siersbaek‐Nielsen (1992)	Buschang et al. (1986)	Schulhof et al. (1977)
Domain 1: Patient selection	Was a consecutive or random sample of patient enrolled? **(Y,N,U)**	N	N	Y	N	N	N	N	N	Y	N
	Was a case control design avoided? **(Y,N,U)**	Y	Y	Y	Y	Y	Y	Y	Y	Y	Y
	Did the study avoid inappropriate exclusions? **(Y,N,U)**	Y	Y	Y	Y	N	N	Y	U	N	N
	Could the selection of patients have introduced bias? **(H,L,U).**	H	H	L	H	H	H	H	H	H	H
	Concerns regarding applicability: Is there concern that the included patients do not match the review question? **(H,L,U).**	L	L	L	L	L	L	L	L	L	L
Domain 2: Index test	Were the index test results interpreted without knowledge of the results of the reference standard? **(Y,N,U).**	N	U	N	Y	N	N	N	N	N	N
	If a threshold was used, was it pre‐specified? **(Y,N,U)**	U	Y	N	N	U	U	Y	U	U	U
	Could the conduct or interpretation of the index test have introduced bias? **(H,L,U)**	H	U	U	L	H	U	L	U	U	L
	Concerns regarding applicability: Is there concern that the index test, its conduct, or interpretation differ from the review question? **(H, L, U).**	L	L	L	L	L	L	L	L	L	L
Domain 3: Reference standard	Is the reference standard likely to correctly classify the target condition? **(Y, N, U)**	Y	Y	Y	Y	Y	Y	Y	U	Y	Y
	Were the reference standard results interpreted without knowledge of the results of the index test? **(Y, N, U)**	U	U	U	U	U	U	Y	U	U	Y
	Could the reference standard, its conduct, or its interpretation have introduced bias? **(H, L, U)**	U	L	L	L	U	U	L	U	U	L
	Concerns regarding applicability: Is there concern that the target condition as defined by the reference standard does not match the review question? **(H,L,U)**	L	L	L	L	L	L	L	L	L	L
Domain 4: Flow and timing	Was there an appropriate interval between index test(s) and reference standard? **(Y,N,U)**	Y	Y	Y	Y	U	Y	Y	Y	Y	Y
	Did all patients receive a reference standard? **(Y,N,U)**	N	Y	Y	Y	Y	U	Y	Y	Y	Y
	Did patients receive the same reference standard? **(Y,N,U)**	Y	Y	Y	Y	Y	Y	Y	Y	Y	Y
	Were all patients included in the analysis? **(Y,N,U)**	N	Y	Y	Y	Y	U	Y	Y	U	Y
	Could the patient flow have introduced bias? **(H,L,U)**	L	L	L	L	L	L	L	L	L	L

*Note*: Yes (Y), no (N), unclear (U). Risk: Low (L)/High (H)/Unclear (U).

**TABLE 6:** Quality assessment GRADE

	Random sequence generation	Allocation concealment	Blinding of participants and personnel	Blinding of outcome assessment	Incomplete outcome data	Selective reporting	Other bias
Auconi et al. (2014)	H	H	H	H	L	L	U
Scala et al. (2012)	H	H	H	H	L	H	L
Turchetta et al. (2007)	L	H	H	H	L	L	H
Arias et al. (2006)	H	U	H	U	H	L	U
Chen et al. (2005)	H	H	H	H	L	L	H
Abu Alhaija and Richardson (2003)	H	H	H	H	L	L	L
Rudolph et al. (1998)	H	H	H	H	L	L	U
Solow and Siersbaek‐Nielsen (1992)	H	H	H	H	L	L	H
Buschang et al. (1986)	L	H	H	H	L	L	U
Schulhof et al. (1977)	H	H	H	H	L	H	H

*Note*: H, High; L, Low; U, Unclear.

### 4.2 Synthesis of results

The results collected from the included studies were based on levels of prediction of measurements made on clinical and cephalometric data, from which multivariate analyses, prediction based on equations, correlation analysis, univariate statistical analyses and computational methods were performed, allowing the design of methods of prediction for maxillary and mandibular growth. Studies were considered heterogeneous and quantitative data were not comparable, so a meta‐analysis was not considered appropriate.

## 5 DISCUSSION

### 5.1 Summary of evidence

The scarce available evidence suggests that there are few predictors to estimate craniofacial growth in class II and III malocclusions. In general, all predictors were designed based on cephalometric and clinical data and with the following predictors: mathematical equation, computerized structural superimposition, network and computational modeling, cluster analysis, software methods and Fishman method.

This systematic review is based on studies with a low to moderate level of evidence according to GRADE and QUADAS‐2, suggesting that there are few predictors with adequate methodological quality. However, of all predictors analyzed, Fishman could be a recommended method of more individualized prediction, based on skeletal maturation by the evaluation of hand‐wrist radiograph (Turchetta et al., 2007) on the basis of maxillary and mandibular angular estimates for classes I and II and for Class III mandibular group estimates. In addition, the method proposed by Buschang et al. (1986), presents an approach based on a polynomial model, which would provide estimates to describe the average size, speed and acceleration, reducing the required longitudinal cephalometric data.

### 5.2 Quality of the evidence

Ten studies were included for qualitative analysis in this systematic review. Based on their design, all the articles were cohort studies, which indicates that there was a follow‐up in the growth of the subjects to adequately design the prediction systems. All articles presented a high risk of bias when analyzed with GRADE, although the studies conducted by Buschang et al. (1986), Turchetta et al. (2007), Abu Alhaija and Richardson (2003), presented a better methodological quality, particularly in the random sequence generation, allocation concealment and other bias domains compared to the rest of the studies.

When the QUADAS‐2 tool was considered to determine the predictor accuracy, the risk of bias was low to moderate. Most of studies presented biases in some domains of the QUADAS‐2 tool. We found problems in most of the studies, as they did not adequately describe a randomly distributed sample, in the interpretation of the index test and the shielding of the researchers when the test results were interpreted, although all of them coincided with the review question in relation to the index test and reference standard.

### 5.3 Potential biases in the review process

For the development of this SR, every effort was made to limit the presence of biases in the article selection process by consulting the largest number of electronic databases and without language limitation. In addition, the search included the literature published until 2019. No study included the calculation of the sample size, and in general, the sample size was small in most of the studies. Age distribution included growing patients in all the studies analyzed. Of these, three studies consisted only of female subjects (Auconi et al., 2014; Chen et al., 2005; Scala et al., 2012) and one only of male subjects (Buschang et al., 1986), which could limit the interpretation of the results to growing patients in the general population .

### 5.4 Predictors of growth for class II malocclusions

Five studies proposed predictors of growth for subjects with class II malocclusions. Three articles designed predictors based on mathematical models (Arias et al., 2006; Buschang et al., 1986; Rudolph et al., 1998) and a study in computerized structural superimposition (Solow & Siersbaek‐Nielsen, 1992) that would determine the maxillary longitudinal growth (ss‐pm). Of these, the studies by Buschang et al and Turchetta et al presented a lower risk of bias according to GRADE and QUADAS‐2. The predictor based on a mathematical model proposed by Buschang et al. (1986), would consider maxillary and mandibular growth (Mandibular length (Ar‐Po)/Length of ramus height (Ar‐Go)). Finally, Turchetta et al. (2007), proposed the Fishman prediction system, based on skeletal maturation determined by the evaluation of hand‐wrist radiograph with high precision in the short and long term.

The findings made would allow clinicians to determine and predict stability in treatment. Although the systems proposed by Buschang et al. (1986) and Turchetta et al. (2007) could be good predictors, there would be other factors influencing the success of treatment in class II malocclusions and not considered by them. Based on the above, there is evidence suggesting that the risk of recurrence or lack of stability in class II treatment could be attributed to a severe pre‐treatment sagittal relationship (greatly increased overjet and a class II cusp ratio in molars and canines) and even the timing of the treatment would not have a greater influence on recurrence (Wins et al., 2016), which would not be considered in the proposed prediction systems.

### 5.5 Predictors of growth for class III malocclusions

Six articles designed growth predictors for class III malocclusions. Some studies included the design of predictors using resources such as: Computational Modeling (Auconi et al., 2014), Network Modeling (Scala et al., 2012), Cluster analysis (Abu Alhaija & Richardson, 2003) and Software methods (Schulhof et al., 1977), all constructed from cephalometric data. Meanwhile, other prediction systems were designed based on cephalometric analysis (Ricketts analysis, the Johnston grid system, and the Fishman) (Turchetta et al., 2007) and the use of a linear equation based on a mathematical model to predict mandibular growth (Chen et al., 2005). Of these, the predictors designed in the Turchetta and Abu Alhaija studies (Abu Alhaija & Richardson, 2003; Turchetta et al., 2007) presented the lowest risk of bias according to the GRADE and QUADAS‐2 tools.

These predictors could have clinical relevance in subjects who will undergo orthodontic and/or orthopedic treatment with the objective of defining the beginning of the camouflage treatment during growth or waiting until the growth is complete to plan an orthodontic‐surgical treatment (Ghiz et al., 2005). Turchetta et al. (2007), concluded that Fishman's method could be the best in the short and long term. This method is based on maturational age determined by hand‐wrist radiograph. The percentages of total growth completed are considered instead of linear growth in absolute terms, several facial linear measurements are applied to construct a prediction. According to the authors, when evaluating maturational development instead of chronological age, physiological variability among children at the same chronological age is reduced (Turchetta et al., 2007), which would increase the accuracy. In the study performed by Abu Alhaija and Richardson (2003), 3 clusters were formed: long facial types (cluster I), short (severe class III discrepancy or cluster II) and intermediate (moderate intermaxillary discrepancy or cluster III). The percentage of discrimination was 80% when DFA was performed (discriminant function analysis), which was satisfactory, but when the analyses were performed in the groups separately, the results varied for cluster I with a good or bad result in 92%, 85% cluster II and 100% cluster III. The authors concluded that using this predictor could identify good and bad growers based on the change in Wits measurements with projection in the bisector of the maxillary/mandibular plane, where the cut‐off point between good and bad growers would be a Wits value of 2.5 mm (upper limit of the 95% confidence interval) (Abu Alhaija & Richardson, 2003).

The other four studies (Auconi et al., 2014; Chen et al., 2005; Scala et al., 2012; Schulhof et al., 1977), presented a high risk of Schulhof et al. (1977), designed a predictor (=V CN/SD × V), and concluded that the sum of the deviations of the measurements of the molar ratio, cranial deflection, ramus position and porion location, would be important in the prediction of class III in cases of greater mandibular growth. Chen et al. (2005), formulated a linear equation to determine the mandibular growth potential from cephalometric data and hand x‐rays to obtain skeletal maturation with an accuracy that implied an average error of 1.45 mm, lower when compared to other methods. A limitation of this study was that the population consisted only of Japanese women. Scala et al. (2012), applied a network modeling in 532 class III young females, concluding that during the growth of class III malocclusion, the characteristics of vertical and sagittal growth (SN‐CoGn, PP‐PM) would be central in the interactive network of system components (orofacial growth controlling nodes). The limitations of the study were: a high risk of bias, a sample consisting only of female patients and a lack of characterization of class III patients, that is, to determine if they had a greater mandibular growth, maxillary hypoplasia or combination of both. Auconi et al. (2014), applied a combination of computational techniques, such as Fuzzy clustering and Network analysis from cephalometric data of 429 growing women. They concluded that four parameters would provide the best phenotypic grouping: Co‐A, Co‐Gn, SNB and P22 (combination of SN GoGn and ArGoMe angles).

Although all the predictors analyzed in this review were constructed from the follow‐up of growing patients and the data were obtained from the clinic, cephalometry and/or radiographs, the genetic factor should be considered for future studies. The new findings could explain the genetic susceptibility to the class III phenotype with mandibular prognathism when there is presence of GHR and FGF polymorphisms, and could also explain the CA genotype of P561T with greater mandibular length (Co‐Gn) (Bayram et al., 2014) .The natural progression of class III has not been accurately tested yet, since most of the evidence is based on case–control studies that cannot yet establish an association between genetic variation and class III malocclusion (Cruz et al., 2017), which has not been evaluated in the studies analyzed in this review.

### 5.6 Predictors of growth in treated patients

There are proposals of predictors to determine the success of treatment in growing subjects, however, these studies were not included in this review because they considered the intervention of subjects under orthopedic and/or orthodontic treatment. Despite the evidence in this topic, there would be no consensus predictor, since the differences given in the sample collection, the characterization of the subjects (excessive mandibular growth, lack of maxillary growth, combination of both and hypo or hyperdivergent growth pattern), long‐term follow‐up and different classification criteria make this difficult. In the study by Ghiz et al. (2005), a logistic regression model was developed to identify the dentoskeletal variables responsible for the outcome of treatment success in subjects with class III malocclusion who underwent orthopedic treatment to perform a maxillary protraction. They concluded that in growing class III patients with an advanced mandibular position, a smaller ramus length, increased mandibular length and an obtuse gonial angle could be unsatisfactorily associated with the results of treatment after pubertal growth. Kim et al. (2009), using the “feature wrapping (FW)” method in class III subjects treated in a first and second phase treatment using the SVM and SFS algorithms, obtained better accuracy with AB‐MP (AB at the angle of the mandibular plane) and A to N perpendicular (mm), and they were the most accurate cephalometric predictors with the FW and DA (discriminant analysis) methods, with an accuracy of 97.3%. They established that a low AB‐MP value would indicate a hyperdivergent skeletal pattern and a severe degree of prognathism and the A‐N perp predictor would describe the anteroposterior position of the maxilla due to the presence of classes III by a retrusive A point. Most studies that attempt to predict craniofacial growth in intervened or untreated subjects establish their predictions through statistical methods and do not design predictors to be applied in orthodontic practice. Among these predictive variables, Singer et al. (1987) stated that the clinical presence of a deep mandibular antegonial notch would be indicative of decreased mandibular growth and vertical mandibular growth; Rossouw et al. (1991) suggested that in class I and III malocclusions, the frontal sinus surface (in mm^2^) would be an indicator to predict increased mandibular growth in subjects with a larger frontal sinus. Arntsen and Sonnesen (2011), showed that fusion abnormalities in the cervical spine would be associated with a greater mandibular sagittal relationship, mandibular retrognathia, greater mandibular inclination and an extended head posture; for class III malocclusions, Yang and Kim (1995) presented the sum of Björk, the gonial angle and the occlusal plane to the angle of the AB plane; Ko et al. (2004), the incisor inferior to the angle of the mandibular plane and Baccetti et al. (2004), the mandibular ramus, angle of the skull base and angle of the mandibular plane.

### 5.7 Limitations

The limited evidence and risk of bias found in most articles constitutes a limitation of this SR. Although all the studies designed predictors based on cephalometric data, these were not similar and most authors proposed different types of predictors. In spite of the similarity of the design in most of the articles, (all were cohort studies), the heterogeneity of the methodology to propose prediction models does not allow comparisons between them, and neither does the difference in the system of prediction. In addition, the risk of bias present in most of the studies analyzed using the GRADE and QUADAS‐2 tools would be mainly due to the absence of randomization of the sample, shielding and interpretation of the index test, which suggests improving these items in future research.

It was not possible to propose a single method, since most of the predictors designed in the studies were established from multiple cephalometric variables, and there is no standardization of the points, angular and/or linear measurements, also considering the heterogeneity of the designs (prospective or retrospective cohort) and characterization of malocclusions, which makes it even more difficult to establish any comparison. Despite this, and based on the findings made in this review, it is possible to suggest that the predictors for the growth of classes II and III proposed by Buschang et al. (1986) and Turchetta et al. (2007) could be useful in orthodontic practice as their methodological quality is better.

Given the heterogeneity of the methodology used in the studies, in the designs of the predictors, number of patients and distribution by gender, it was not possible to perform a meta‐analysis.

## 6 CONCLUSIONS

Predicting growth is one of the most relevant challenges in the field of craniofacial growth and development, as it would allow the planning and prediction of timing and prognosis of first and second phase treatments in orthodontics.

From the findings made in this systematic review, it is possible to conclude the following:

- The available evidence from studies that design class II and III predictors is scarce and their methodological quality in general is low to moderate.
- There is no consensus to establish a single predictor, since the designs of the studies are heterogeneous, the extraction of data from the studies was not standardized and in general they do not characterize the patients.
- More cohort studies with a higher level of evidence are suggested, with more homogeneous designs and standardized methods to extract the data from the clinic, radiographs and cephalometrics methods.

## CONFLICT OF INTEREST

The authors report no conflicts of interest.

## AUTHOR CONTRIBUTIONS

All authors read and approved the manuscript and their contribution to the study was: Jiménez‐Silva Antonio: Search strategy, selection of studies, extracting studies from databases, data collection, analysis of studies according to the PICO format. Manuscript preparation. Carnevali‐Arellano Romano: Search strategy, selection of studies, data collection. Analysis of studies according to the PICO format. Vivanco‐Coke Sheilah: Search studies in database, preparation of summary tables, diagrams and determine the quality of the evidence from selected studies. Tobar‐Reyes Julio: Supervision and manuscript correction. Translation of the manuscript and determination of the quality of evidence of the selected studies. Araya‐Díaz Pamela: Supervision and manuscript correction. Translation of the manuscript. Third researcher who collaborated in the selection of articles. Palomino‐Montenegro Hernán: Supervision and manuscript correction. Translation of the manuscript.

## Data Availability

The data that support the findings of this study are available in [repository name] at [URL/DOI], reference number [reference number]. These data were derived from the following resources available in the public domain: [list resources and URLs].

## APPENDIX A.

**TABLE A1:** Search strategy and terms used for the search

Database and limits	Search strategy and terms
PubMed (*n* = 929) Limits: Publication date: Until 30 April 2019	((craniofacial growth predictor OR craniofacial growth latency OR craniofacial growth [tiab] OR dentofacial latency OR growth predictor OR growth latency)) AND (sagittal jaw relation growth OR class II malocclusion growth OR class III malocclusion growth OR skeletal class II growth OR skeletal class III growth OR facial growth OR sagittal jaw relation growth OR sagittal development growth OR jaw relation growth OR skeletal discrepancy growth OR class II skeletal pattern OR class III skeletal pattern growth))
Cochrane library (*n* = 246) Limits: Database: Trials, Publication date: Until April 2019‐	Prediction OR predicting OR predictor OR growth predictor OR latency AND class II malocclusion growth OR class I malocclusion growth OR class III malocclusion growth OR skeletal class II growth OR skeletal class III growth OR facial growth OR sagittal jaw relation growth OR jaw relation OR skeletal discrepancy OR class II skeletal pattern growth OR class III skeletal pattern growth
EBSCOhost (*n* = 261) *Dentistry & Oral Sciences Source* *Academic Search Ultimate* *Medline* Academic publications: Publication date: 1934–2019.	Craniofacial growth trend OR growth pattern tiab OR growth direction tiab OR craniofacial growth pattern OR dentofacial growth predictor OR craniofacial latency OR craniofacial growth latency OR dentofacial latency OR craniofacial growth predictor OR prediction OR predicting OR predictor OR growth predictor OR latency OR growth latency AND sagittal jaw relation OR class II malocclusion OR class III malocclusion OR skeletal class II OR skeletal class III OR facial growth OR sagittal jaw relation OR sagittal development OR jaw relation OR skeletal discrepancy OR class II skeletal pattern OR class III skeletal pattern OR craniofacial relationship OR class II tiab OR class III tiab OR maxillo‐mandibular relationship OR dental arch discrepancy OR class II morphology OR class III morphology OR sagittal skeletal discrepancies
Scopus (*n* = 221) Document type: Article type Date range: Until 2019	Growth indicator OR prediction OR predictor OR latency OR growth latency OR craniofacial growth pattern OR dentofacial growth predictor OR craniofacial latency OR craniofacial growth latency OR dentofacial latency OR craniofacial growth predictor AND class II malocclusion OR class III malocclusion OR class II OR class III OR maxillo‐mandibular relationship
Embase (*n* = 308) Publication years: 1966–2019 Publication type: Article Study type: Humans Age: Child (1–12), preschool child (1–6), school child (7–12), adolescent, young adult.	Predictor OR indicator OR latency OR craniofacial pattern OR craniofacial growth predictor OR craniofacial growth pattern OR growth latency OR growth predictor OR growth pattern OR dentofacial pattern OR prediction AND class II malocclusion OR class III malocclusion OR class II OR class III OR skeletal class III
Bireme (*n* = 109) Clinical point of view: Prognosis, prediction, diagnosis. Publication date: Until 2019	(tw:(predictor OR predicting OR prediction OR growth predictor OR latency OR growth latency OR growth indicator OR predictor OR indicador de crecimiento craneofacial OR predictor de crecimiento craneofacial OR latencia OR latencia de crecimiento OR predictor de crecimiento)) AND (tw:(class II malocclusion OR class III malocclusion OR class II OR class III OR dental malocclusion OR clase II esqueletal OR clase III esqueletal OR maloclusión de clase II OR maloclusion de clase III))
Scielo (*n* = 132) Publication date: Until 2019 Type of study: Article	(predictor OR growth predictor OR latency OR pattern OR trend OR indicador) AND (class III malocclusion OR class II OR class III OR clase III OR skeletal class III)
Lilacs (*n* = 30)	(tw:(predictor OR predicting OR prediction OR growth predictor OR latency OR growth latency OR growth indicator OR predictor OR indicador de crecimiento craneofacial OR predictor de crecimiento craneofacial OR latencia OR latencia de crecimiento OR predictor de crecimiento)) AND (tw:(class II malocclusion OR class III malocclusion OR class II OR class III OR dental malocclusion OR clase II esqueletal OR clase III esqueletal OR maloclusión de clase II OR maloclusion de clase III))
Science direct (*n* = 209) Publication date: All years	Craniofacial growth predictor OR predictor OR predicting OR growth predictor OR growth trend OR craniofacial growth trend AND class III malocclusion OR class II malocclusion OR class II craniofacial pattern OR class III craniofacial pattern OR class II OR class III

**TABLE A2:** Studies retrieved in full text and excluded from the review

First author and year	Reason for exclusion
Radalj Miličić et al. (2018)	Predictive measures in class III patients to determine rotational pattern
Engel et al. (2016)	It does not predict skeletal class or amount/type of growth, it only determines that CVM does not predict peak growth in girls.
Masoud et al. (2015)	Study proposes predictor, but does not discriminate between classes II and III and evaluates only vertical growth.
Salehi et al. (2012)	Iranian language
Murata (2009)	Does not propose predictor
Hunter et al. (2007)	No difference by skeletal class
Reyes et al. (2006)	It does not propose a predictor. Compares class III with class I/II
Chvatal et al. (2005)	Does not propose predictor. No difference by skeletal class
Flores‐Mir et al. (2004)	Systematic Review.
Hilger et al. (2003)	Predictions of future mandibular shapes and size/No difference by skeletal class
Kolodziej et al. (2002)	Craniofacial growth prediction. It does not differentiate class II and class III.
Arat et al. (2001)	Does not propose predictor. No difference by skeletal class
Zhou et al. (2000)	Article in Chinese language/It is not possible to determine whether the prediction system was in class II or III
Aki et al. (1994)	Does not propose predictor. No difference by skeletal class
Snodell et al. (1993)	Predictor of growth in class I
Rossouw et al. (1991)	It establishes an association between frontal sinus size and mandibular growth.
Todd and Mark (1981)	It is not a primary study/No difference by skeletal class.
Hirschfeld and Moyers (1971)	It is not a primary study/No difference by skeletal class.
